# Consensus on criteria for acromegaly diagnosis and remission

**DOI:** 10.1007/s11102-023-01360-1

**Published:** 2023-11-03

**Authors:** Andrea Giustina, Nienke Biermasz, Felipe F. Casanueva, Maria Fleseriu, Pietro Mortini, Christian Strasburger, A. J. van der Lely, John Wass, Shlomo Melmed, Acromegaly Consensus Group, Giuseppe Banfi, Ariel Barkan, Albert Beckers, Martin Bidlingmaier, Cesar Boguszewski, Thierry Brue, Michael Buchfelder, Philippe Chanson, Sabrina Chiloiro, Annamaria Colao, Eva Coopmans, Daniela Esposito, Diego Ferone, Stefano Frara, Mônica Gadelha, Eliza B. Geer, Ezio Ghigo, Yona Greenman, Mark Gurnell, Ken Ho, Adriana Ioachimescu, Gudmundur Johannsson, Jens Otto Jørgensen, Ursula B. Kaiser, Niki Karavitaki, Laurence Katznelson, Stephen Lamberts, Marco Losa, Anton Luger, Raúl Luque, Pietro Maffei, Mónica Marazuela, Sebastian Neggers, Alberto Pereira, Luca Persani, Stephan Petersenn, Martin Reincke, Roberto Salvatori, Susan N. Samson, Katharina Schilbach, Ilan Shimon, Stylianos Tsagarakis, Maria Chiara Zatelli

**Affiliations:** 1 San Raffaele Vita-Salute University and IRCCS Hospital, Milan, Italy; 2 Leiden University Medical Center, Leiden, The Netherlands; 3 Santiago de Compostela University, Santiago de Compostela, Spain; 4 Oregon Health and Science University, Portland, OR USA; 5 Charité Universitätsmedizin, Berlin, Germany; 6 Erasmus University Medical Center, Rotterdam, The Netherlands; 7 Churchill Hospital, Oxford, UK; 8 Cedars-Sinai Medical Center, 8700 Beverly Blvd, NT 2015, Los Angeles, CA 90048 USA

**Keywords:** Acromegaly, Growth hormone, Insulin-like growth factor I, Assays, Diagnosis, Remission criteria

## Abstract

**Purpose:**

The 14th Acromegaly Consensus Conference was convened to consider biochemical criteria for acromegaly diagnosis and evaluation of therapeutic efficacy.

**Methods:**

Fifty-six acromegaly experts from 16 countries reviewed and discussed current evidence focused on biochemical assays; criteria for diagnosis and the role of imaging, pathology, and clinical assessments; consequences of diagnostic delay; criteria for remission and recommendations for follow up; and the value of assessment and monitoring in defining disease progression, selecting appropriate treatments, and maximizing patient outcomes.

**Results:**

In a patient with typical acromegaly features, insulin-like growth factor (IGF)-I > 1.3 times the upper limit of normal for age confirms the diagnosis. Random growth hormone (GH) measured after overnight fasting may be useful for informing prognosis, but is not required for diagnosis. For patients with equivocal results, IGF-I measurements using the same validated assay can be repeated, and oral glucose tolerance testing might also be useful. Although biochemical remission is the primary assessment of treatment outcome, biochemical findings should be interpreted within the clinical context of acromegaly. Follow up assessments should consider biochemical evaluation of treatment effectiveness, imaging studies evaluating residual/recurrent adenoma mass, and clinical signs and symptoms of acromegaly, its complications, and comorbidities. Referral to a multidisciplinary pituitary center should be considered for patients with equivocal biochemical, pathology, or imaging findings at diagnosis, and for patients insufficiently responsive to standard treatment approaches.

**Conclusion:**

Consensus recommendations highlight new understandings of disordered GH and IGF-I in patients with acromegaly and the importance of expert management for this rare disease.

## Introduction

Acromegaly caused by a growth hormone (GH)-secreting pituitary adenoma can deleteriously affect patient quality of life (QOL) and mortality if not diagnosed early and properly treated [1]. Insulin-like growth factor (IGF)-I and GH measurements are commonly used as biochemical markers of disease activity for diagnosis and follow-up of acromegaly [2]: IGF-I levels are reflective of GH action on peripheral tissue, primarily in the liver, while GH levels reflect somatotroph adenoma secretory activity.

The first Acromegaly Consensus Conference held in 1999 in Cortina, Italy, concluded that a diagnosis of acromegaly is excluded if random GH is < 0.4 µg/L and age- and sex-matched IGF-I is normal, or if GH nadir is < 1 µg/L during 75-g oral glucose tolerance test (OGTT) and IGF-I is normal [3]. Biochemical control after treatment of acromegaly was defined as achieving normal IGF-I and, after surgery, nadir GH < 1 µg/L during OGTT (Table 1).

**Table 1:** Evolution of criteria for acromegaly diagnosis and evaluation of therapeutic efficacy

	Diagnosis	Therapeutic efficacy target
1st Acromegaly consensus [3]	IGF-I elevated for age and sex Confirm with random GH ≥ 0.4 µg/L *or* IGF-I elevated for age and sex Confirm with GH > 1 µg/L during OGTT	IGF-I normalized for age and sex GH < 1 µg/L during OGTT
7th Acromegaly consensus [4]	IGF-I elevated for age and sex *and* Random GH elevated	Random GH < 1 µg/L GH < 0.4 µg/L during OGTT
Endocrine society guidelines [5]	IGF-I elevated for age Confirm with GH > 1 µg/L during OGTT	IGF-I normalized for age Random GH < 1 µg/L
14th Acromegaly consensus (this publication)	IGF-I > 1.3 × ULN for age *and* Characteristic clinical signs of disease For equivocal results, IGF-I measurements can be repeated, and OGTT might additionally be useful	IGF-I normalized for age

*GH* growth hormone; *IGF-I* insulin-like growth factor I; *OGTT* oral glucose tolerance test; *ULN* upper limit of normal

Revisiting this issue in 2010, the 7th Acromegaly Consensus Conference recommendations included two changes [4]: (1) OGTT is not required for diagnosis if IGF-I and GH levels are clearly elevated; and (2) definition of biochemical control could be adjusted to nadir GH < 0.4 µg/L during OGTT if using newer, ultrasensitive assays. In 2014, guidelines from the Endocrine Society further adjusted these criteria [5]. They recommended using IGF-I normalized to age but not sex for the diagnosis of acromegaly, confirmed by lack of suppression of GH < 1 µg/L during OGTT if necessary, and to use age-normalized IGF-I and random GH < 1.0 µg/L as a therapeutic goal.

Following on studies underscoring the challenges of uniformly applying results of GH and IGF-I assays in the clinic [6, 7], the 14th Acromegaly Consensus Conference held in 2022 in Stresa, Italy, once again revisited the question of how to define biochemical criteria for acromegaly diagnosis and evaluation of therapeutic efficacy. Key points from the discussions are presented here and are summarized in Table 2.

**Table 2:** Key recommendations

**Overall**
Referral to a multidisciplinary pituitary center should be considered for patients with equivocal biochemical, pathology, or imaging findings at diagnosis, and for patients insufficiently responsive to standard treatment approaches.
**Diagnostic assessment**
For all biochemical assessments, clinicians should know which assay is being used, which factors influence its performance, how normal ranges are obtained, and how it has been calibrated and validated.
In a patient with typical clinical signs and symptoms of acromegaly, IGF-I > 1.3×ULN for age confirms the diagnosis. Random GH measured after overnight fasting may be useful for informing prognosis, but is not required for diagnosis. For patients with equivocal results, IGF-I measurements can be repeated using the same validated assay, and OGTT might additionally be useful.
* IGF-I and GH Assays*
Well-validated IGF-I assays should be calibrated to the current international standard (02/254). Age-stratified reference ranges should be based on adequate numbers of subjects; sex-stratified reference ranges are likely not required beyond puberty if the normative population is sufficiently large.
BMI might influence normal IGF-I ranges, such that patients with high BMI have lower IGF-I levels for their age group. Nutritional, genetic, metabolic, and hepatic factors can also impact IGF-I concentrations, often inducing states of GH resistance.
There is currently no evidence that IGF-I measurement by mass spectrometry is superior to measurement by immunoassay.
Calibration to the current international standard for GH (98/574) should be standard with immunoassays but has not been validated for mass spectrometry.
* OGTT*
If OGTT is performed, 75 g glucose should be administered after fasting, and GH nadir assessed after 30, 60, 90, and 120 min.
BMI-based GH nadir cutoffs of < 0.4 µg/L for BMI < 25 kg/m^2^ and < 0.2 µg/L for BMI ≥ 25 kg/m^2^ can be considered.
Cessation of oral estrogen therapy 4 weeks prior to OGTT may avoid its effects on the GH axis.
OGTT can be safely performed among patients with impaired glucose tolerance or type 2 diabetes mellitus. However, in patients with uncontrolled diabetes, both random and post-OGTT GH levels should be interpreted with caution.
Measurement of basal and 120-minute glucose levels during OGTT is useful for detecting disturbances in glucose homeostasis.
* Clinical, Imaging, and Pathology Assessments*
A careful history and physical exam is required as it will often reveal unequivocal signs and symptoms related to local mass effect or secondary features of GH and IGF-I hypersecretion.
Gadolinium-enhanced pituitary MRI should be performed in patients at diagnosis using high-quality, high-resolution equipment.
Reporting should include information on invasion into surrounding structures based on modified Knosp grade.
Equivocal diagnosis of acromegaly associated with pituitary microadenomas should be referred for review by an experienced neuroradiologist before considering further imaging studies.
Standard pathology reporting should include immunohistochemistry assessment for pituitary hormones. Transcription factors can be used to define adenoma lineage and further characterize adenoma cell type when not classifiable on hormone expression alone.
Clinical implications of the 2022 WHO classification suggesting that pituitary adenomas could also be called pituitary neuroendocrine tumors remain unclear and the ongoing ramifications for acromegaly patients are not apparent.
* Diagnostic delay*
Prolonged exposure to excess GH with diagnostic delay leads to increased comorbidity and mortality risks with decreased QOL, and could lead to reduced treatment success and increased need for additional therapy.
Strategies aimed at reducing diagnostic delay should be implemented worldwide as they may reduce short-term and long-term morbidity and positively impact QOL.
All patients with a newly diagnosed pituitary mass should undergo IGF-I measurement.
Although widespread screening in the general population is not warranted, IGF-I screening could be considered in individuals with classical signs, symptoms, and comorbidities of acromegaly including acral enlargement and orofacial changes, particularly if these occur in conjunction with unexplained systemic manifestations such as sleep apnea or ventricular hypertrophy.
A systematic approach should be implemented among healthcare practitioners to increase awareness about acromegaly. Outreach strategies in collaboration with patient advocacy groups such as for other rare diseases could also help promote earlier referral for diagnostic testing.
**Criteria for remission**
The term “remission” indicating that active disease cannot be detected even if it might still be present is the most accurate descriptor for biochemical treatment outcome goals in acromegaly.
Although biochemical remission is the primary assessment of treatment outcome, it is not the only goal of treatment in acromegaly. In all cases, biochemical findings should be interpreted within the clinical context of acromegaly signs and symptoms.
Maintaining serum IGF-I level in the mid to upper half of the age-related reference range could be considered in clinically controlled patients to avoid induction of GH deficiency.
* Postoperative remission*
There are no definitive studies on the optimal assessment for postoperative remission, nor of the timing of its evaluation.
IGF-I should be measured at 12 weeks after surgery to determine postoperative biochemical remission. Early random GH assessment on day 1–14 and comparison with preoperative GH can inform the degree of adenoma removal and subsequent longer-term remission.
OGTT assessment may provide further predictive value.
In patients treated with preoperative SRL, assessment should be repeated at 3–6 months to confirm remission.
* Remission With Adenoma-Directed Medical Therapy*
For patients treated with injectable SRL, IGF-I level measurement in the last week before the next injection should be used to determine a need for dose titration or consideration of alternative treatment options if normalization is not achieved.
For patients treated with oral SRL administered daily, assessment of IGF-I for the purposes of dose titration should be done after at least 2 weeks of treatment.
Timing of IGF-I assessment is not critical for patients treated with cabergoline administered in more than once-weekly intervals.
With all of these agents, random GH assessment is not likely to provide additional information in all patients but could be considered for symptomatic patients with IGF-I levels at the higher end of the ULN.
* Remission With Peripherally Directed Medical Therapy*
For patients treated with medical therapy that targets the GH receptor or the estrogen receptor, efficacy assessment is limited to IGF-I normalization.
With these agents, GH assessment is not informative and should not be performed.
**Follow up**
Follow up assessments should consider biochemical evaluation of treatment effectiveness, imaging studies evaluating residual/recurrent adenoma mass, and clinical signs and symptoms of acromegaly and its complications and comorbidities.
* Biochemical*
Within the first postoperative year, IGF-I measurements every 3–6 months may be appropriate to confirm remission, and then every 6–12 months to monitor for potential recurrence. OGTT might be helpful in evaluating patients with borderline IGF-I levels and clinical signs of disease activity.
For patients who did not achieve postoperative remission and who are treated with adjuvant SRL, IGF-I should be assessed 3 months after initiation/dose adjustment of injectable SRL and 2–4 weeks after initiation/dose adjustment of oral SRL to establish an optimal dosing regimen, and then every 6–12 months thereafter once biochemical control is achieved. Random GH might be helpful in select cases where evaluation of adenoma behavior is a concern.
As pegvisomant and cabergoline have a shorter half-life than injectable SRL, IGF-I should be assessed every 1–3 months after initiation/dose adjustment to establish the dosing regimen, and then every 6–12 months thereafter.
In patients receiving medical therapy as a bridge until radiotherapy effect is seen, IGF-I should be assessed at the intervals appropriate for the medical therapy used. With sustained decline of IGF-I within the target range, treatment can be paused at least once each year depending on rapidity of the IGF-I decline to test for the onset of radiation-induced remission.
For all patients, ideally, the same well-validated IGF-I assay should be used for all assessments. New or persistent elevations in IGF-I levels should be interpreted within the context of the individual clinical scenario and account for factors that could affect results such as pregnancy, estrogen use, starvation, and metabolic changes.
* Imaging*
The same standards for imaging and results reporting should be used in follow up as in diagnosis. MRI should be performed at 3–6 months postoperatively and used as baseline for further assessments. MRI should be performed upon signs of biochemical or clinical disease progression, and when a change in therapeutic modality is considered, such as prior to a second surgery or radiotherapy. An individualized approach to MRI is appropriate for patients treated with pegvisomant based on country-specific labeling requirements, as well as for those with genetic markers or prior imaging suggestive of highly aggressive disease.
* Clinical assessment*
This Workshop endorsed evaluation and treatment of disease comorbidities according to prior consensus recommendations. The need for assessment of common comorbidities, such as hypopituitarism, obstructive sleep apnea, and vertebral fractures depends on clinical symptoms and adenoma behavior, and follow up according to accepted guidelines was recommended.
There was no consensus at this Workshop on whether colonoscopy should be performed in all acromegaly patients at diagnosis regardless of age. For all other cancers with reported increased frequency in acromegaly, including thyroid cancer, there was consensus that screening be performed according to national/regional guidelines for the general population.
SAGIT and ACRODAT may be useful in current clinical practice for assessing changes in acromegaly disease severity and progression over time. A prospective study measuring a clinically beneficial effect of ACRODAT as a monitoring tool is underway.
* Considerations for second- and third-line treatment selection*
Follow up assessments identify patients more likely to show a favorable response to second- and third-line medical therapy options if needed.
Results of follow up assessments can also be used to identify patients who might benefit from treatment options that have an improved safety profile or more convenient dosing regimen.

*BMI* body mass index; *GH* growth hormone; *IGF-I* insulin-like growth factor I; *MRI* magnetic resonance imaging; *OGTT* oral glucose tolerance test; *QOL* quality of life; *SRL* somatostatin receptor ligand; *WHO* World Health Organization; *ULN* upper level of normal

## Materials and methods

The process for development of consensus recommendations by Acromegaly Consensus Group participants before and during the meeting has been described [8]. Briefly, participants (Table 3) were assigned specific topics related to acromegaly diagnosis and follow-up and conducted comprehensive literature searches for English-language papers published between January 2015 and September 2022. Search terms included “acromegaly” as well as terms associated with each respective topic covered. After brief presentations to the entire group on each topic, breakout groups discussed current practice and recommendations, and a summary of the findings was reported back to the entire group. Consensus recommendations were developed based on all presentations and discussions and all participants voted on each recommendation. After the meeting, members of the Scientific Committee graded both the quality of the supporting evidence and the consensus recommendations based on principles for grading of evidence for guidelines and prior Acromegaly Consensus publications [9–11]. Evidence was graded by strength as very low quality (VLQ), low quality (LQ), moderate quality (MQ), or high quality (HQ), and recommendations were classified as discretionary (DR) or strong (SR) as indicated in Table 4.

**Table 3:** Acromegaly consensus group participants

Giuseppe Banfi (IT), Ariel Barkan (US), Albert Beckers (BE), Martin Bidlingmaier (DE), Nienke Biermasz (NL), Cesar Boguszewski (BR), Marcello Bronstein (BR), Thierry Brue (FR), Michael Buchfelder (DE), Felipe F. Casanueva (ES), Philippe Chanson (FR), Sabrina Chiloiro (IT), Annamaria Colao (IT), Eva Coopmans (NL), Daniela Esposito (SE), Diego Ferone (IT), Maria Fleseriu (US), Stefano Frara (IT), Mônica Gadelha (BR), Eliza B. Geer (US), Ezio Ghigo (IT), Andrea Giustina (IT), Yona Greenman (IS), Mark Gurnell (UK), Ken Ho (AU), Adriana Ioachimescu (US), Gudmundur Johannsson (SE), Jens Otto Jørgensen (DK), Ursula B. Kaiser (US), Niki Karavitaki (UK), Laurence Katznelson (US), Stephen Lamberts (NL), Marco Losa (IT), Anton Luger (AT), Raúl Luque (ES), Pietro Maffei (IT), Mónica Marazuela (ES), Shlomo Melmed (US), Pietro Mortini (IT), Sebastian Neggers (NL), Alberto Pereira (NL), Luca Persani (IT), Stephan Petersenn (DE), Martin Reincke (DE), Roberto Salvatori (US), Susan L. Samson (US), Katharina Schilbach (DE), Ilan Shimon (IS), Christian Strasburger (DE), Stylianos Tsagarakis (GR), A.J. van der Lely (NL), John Wass (UK), Maria Chiara Zatelli (IT)	

**Table 4:** Grading of evidence and recommendations

**Evidence**	Very low quality (VLQ): expert opinion supported by one or few small uncontrolled studies
	Low quality (LQ): supported by large series of small uncontrolled studies
	Moderate quality (MQ): supported by one or few large uncontrolled studies or meta-analyses
	High quality (HQ): supported by controlled studies or large series of large uncontrolled studies with sufficiently long follow-up
**Recommendations**	Discretionary: based on VLQ or LQ evidence
	Strong: based on MQ or HQ evidence

Based on principles for grading of evidence for guidelines and prior Acromegaly Consensus publications [9–11]

## Diagnostic assessment

Accurate measures of IGF-I and GH are critical to the diagnosis of acromegaly. Therefore, clinicians should know which assay is being used, which factors influence its performance, how normal ranges are obtained (SR), and how it has been calibrated and validated.

### IGF-I and GH assays

In a patient with typical clinical signs and symptoms of acromegaly, IGF-I > 1.3 times the upper limit of normal (ULN) for age confirms the diagnosis (MQ). GH measured after overnight fasting may be useful for informing prognosis or complications, but is not required for diagnosis (SR). However, as it is still often used as first line biochemical assessment [12], a need for the use of validated GH assays worldwide is reinforced (SR). For patients with equivocal results, IGF-I measurements can be repeated using the same validated assay, and OGTT might additionally be useful (DR).

Inter-laboratory and inter-assay discrepancies with IGF-I assays are well known [6, 13, 14]; normal reference ranges are specific to each immunoassay, with the greatest differences seen at the highest values [6] (MQ). Well-validated IGF-I should be calibrated to the current international standard (02/254) [15]. Age-stratified reference ranges should be based on adequate numbers of subjects (SR), but sex-stratified reference ranges are likely not required beyond puberty if the normative population is sufficiently large [16] (DR). However, body mass index (BMI) might influence normal IGF-I ranges, such that patients with high BMI have lower IGF-I levels for their age group [16] (MQ). Nutritional, genetic, metabolic, and hepatic factors can also impact IGF-I concentrations, often inducing states of GH resistance [17–20].

Although mass spectrometry largely eliminates interference from IGF binding proteins that might affect immunoassay results, errors can be introduced during protein concentration and sample preparation, and variability is similar to that seen with immunoassay [21] (LQ). There is currently no evidence that IGF-I measurement by mass spectrometry is superior to measurement by immunoassay (LQ).

Variability in GH immunoassay assessments is commonly encountered because of differences in antibody and epitope binding of GH isoforms, and variability may be greatest with higher values [15] (MQ). Calibration to the current international standard for GH (98/574) should be standard with immunoassays [15], but has not been validated for mass spectrometry methodologies and its use in this setting remains somewhat undefined (DR).

### OGTT

GH nadir during OGTT correlates with spontaneous trough inter-pulse GH concentrations [22], which determine the magnitude of IGF-I production [23] (MQ). Thus, glucose-suppressed GH nadir is effectively an indirect assessment of IGF-I and a reflection of preserved GH neuroregulation [24]. However, there is no cutoff for glucose-suppressed GH that definitively excludes a diagnosis of acromegaly (MQ). GH nadirs in healthy adults vary depending on sex, BMI, and estrogen-containing oral contraceptive (OC) use [7], and the range of both spontaneous trough and glucose-suppressed levels in healthy adults can overlap those of acromegaly patients. Thus, glucose-suppressed GH nadirs in acromegaly patients with lower mean 24-hr GH levels can fall into the range of normal adults [25] (VLQ). Furthermore, up to one-third of patients with acromegaly may show a paradoxical increase in GH following OGTT and may demonstrate up to 50% increase or more in GH levels within 120 min after glucose ingestion [26].

In weighing the available evidence, consensus discussions considered that, in most cases, diagnosis is clear without a need for OGTT, and the interpretative difficulties of OGTT therefore outweigh the potential advantages. Thus, the consensus recommended that this test be reserved for patients in whom baseline hormone levels do not clarify the diagnosis (SR).

If OGTT is performed, 75 g glucose should be administered after fasting [27], and GH nadir assessed after 30, 60, 90, and 120 min [7] (SR). BMI-based GH nadir cutoffs can be considered for diagnosis, with < 0.4 µg/L for BMI < 25 kg/m^2^ and < 0.2 µg/L for BMI ≥ 25 kg/m^2^ [7], although this may be assay dependent (DR). As healthy premenopausal females on estrogen-containing OC have higher GH nadirs [7], cessation of oral estrogen therapy 4 weeks prior to OGTT may avoid its effects on the GH axis.

OGTT can be safely performed in patients with impaired glucose tolerance or type 2 diabetes mellitus, with some applying BMI-based cutoffs [28, 29] (DR). However, due to the suppressive effect of hyperglycemia on GH levels [30], particularly in patients with uncontrolled diabetes [31], both random and post-OGTT GH levels should be interpreted with caution. Measurement of basal and 120-minute glucose levels during OGTT is useful for detecting disturbances in glucose homeostasis (MQ).

### Other assays

A rapid decrease in soluble α-Klotho occurs after adenoma surgical resection, correlating with decreases in IGF-I, and associated with normal IGF-I levels in patients with discordantly elevated random GH levels [32] (LQ). Soluble α-Klotho, but not IGF-I, correlated with GH-dependent symptom scores and disease-specific QOL in patients receiving medical therapy [33] (VLQ)]. However, mechanisms driving soluble α-Klotho secretion in acromegaly as well as assay validation and confirmatory studies are required before it can be considered for use as a biochemical marker in clinical practice (SR).

### Clinical examination

A careful history and physical exam in the initial assessment of patients with suspected acromegaly is required as it will often reveal unequivocal signs and symptoms related to local mass effect or secondary features of GH and IGF-I hypersecretion (SR).

Characteristic changes in the face and head, including widening and malocclusion of the jaw and macroglossia, as well as enlarged hands, occur insidiously but are often apparent at initial assessment [2, 17] (HQ). Moreover, due to diagnostic delay, disease comorbidities and complications including hypertension, diabetes mellitus, and kyphosis [34, 35] should not be overlooked (SR). In fact, they are signs of active disease and may be apparent at initial presentation [36] (LQ). (Diagnosis and management of acromegaly comorbidities are extensively discussed in a separate Consensus Statement [8].) Impaired QOL resulting from the clinical and psychological burden of disease may be present at all stages of disease [37] (VLQ).

### Imaging

Gadolinium-enhanced pituitary MRI should be performed in all patients at diagnosis using high-quality, high-resolution equipment, such as 1.5T or 3T scanners, where available, including T1- and T2-weighted fast spin echo sequences, with coronal and sagittal planes in 2–3 mm slice thickness with no or minimal spacing (SR). Reporting should be standardized, and include information on invasion into surrounding structures based on modified Knosp grade [38] (SR). Adenoma dimensions; suprasellar and infrasellar extension; presence of cystic components; and T2 hypo-, iso-, or hyperintensity of the adenoma compared with adjacent temporal lobe can all be used to inform likelihood of treatment response [39–41] (MQ). Given the proven benefits of expert MRI review in patients with Cushing’s disease microadenomas [42], equivocal diagnosis of acromegaly associated with pituitary microadenomas should be referred for review by an experienced neuroradiologist [43] before considering further imaging studies (SR). Very rarely, cross-sectional imaging and measurement of GH releasing hormone (GHRH) may be needed to identify an ectopic GHRH-secreting neuroendocrine tumor [44] (DR).

PET imaging using ^11^ C-methionine as a molecular tracer may be useful when MRI cannot identify an adenoma at initial diagnosis or, more commonly, a residual adenoma in patients with persistent GH hypersecretion following primary therapy [45, 46] (DR). However, limited availability of both the imaging technology and the tracer constrain their use.

### Pathology

Differentiation of somatotroph, lactotroph, and thyrotroph cells in the pituitary is driven by the PIT1 transcription factor. Somatotroph adenomas are defined on pathology based on immunohistochemistry (IHC) GH expression, and adenomas that secrete/express GH and prolactin may also be seen [47] (HQ). Standard reporting should include IHC assessment for pituitary hormones. Transcription factors can be used to define adenoma lineage and further characterize adenoma cell type when not classifiable on hormone expression alone (SR).

Clinicopathologic classification of pituitary adenomas that considers adenoma invasiveness using Knosp grade and sphenoid sinus invasion as well as proliferation by Ki-67 and mitoses can distinguish adenomas with potentially more aggressive behavior [48], and thus identify patients at increased risk for progression [49, 50] (MQ). Somatostatin receptor immunopositivity, granulation pattern, and *AIP* mutation status have been reported to identify patients less likely to respond to somatostatin receptor ligand (SRL) therapy (DR) [51, 52]. Clinical implications of the 2022 WHO classification suggesting that pituitary adenomas could also be called pituitary neuroendocrine tumors remain unclear [53] and the clinical ramifications for acromegaly patients are not apparent [54].

### Effect of diagnostic delay

Signs and symptoms of acromegaly are nonspecific, and there may be a delay of 5–10 years or more between first symptom onset and diagnosis [55, 56] (HQ). The effect is more pronounced in older patients and in women [57, 58] (MQ) likely due to inappropriate attribution of acromegaly symptoms to normal aging and menopause. Prolonged exposure to excess GH with diagnostic delay leads to increased comorbidity and mortality risks with decreased QOL [55, 59, 60] (HQ).

Importantly, delayed diagnosis also allows for continued adenoma growth as well as invasion into the cavernous sinus, both of which limit successful surgical resection, regardless of surgical expertise [61] (HQ). In these patients, adjuvant medical therapy and/or radiotherapy targeted to the residual mass after debulking surgery might be needed [62] (MQ).

Strategies aimed at reducing diagnostic delay should be implemented worldwide as they may reduce short-term and long-term morbidity and positively impact QOL (SR). All patients with a newly diagnosed pituitary mass should undergo IGF-I measurement (SR). Although widespread screening in the general population is not warranted, IGF-I screening could be considered in individuals with classical signs, symptoms, and comorbidities of acromegaly (DR), including acral enlargement and orofacial changes, particularly if these occur in conjunction with unexplained systemic manifestations such as sleep apnea or ventricular hypertrophy [63]. A systematic approach should be implemented among healthcare practitioners to increase awareness about acromegaly. Outreach strategies in collaboration with patient advocacy groups such as for other rare diseases could also help promote earlier referral for diagnostic testing (SR).

## Criteria for remission

Consensus recommendations previously adjusted criteria for therapeutic goals because of improvements in assay sensitivity and our evolving understanding of GH dynamics after glucose suppression [3–5] (LQ). Additionally, by definition, postoperative IGF-I normalization is a function of the reference values used for each respective assay [15] (HQ). Therefore, an absolute biochemical threshold to define postoperative “cure” does not seem feasible (DR). “Biochemical control,” indicating no biochemical evidence of adenoma GH hypersecretion, is similarly imprecise as measures of GH and/or IGF-I attenuation might be delayed despite complete adenoma resection (DR). By contrast, the term “remission” indicates that active disease cannot be detected even if it might still be present. This was deemed the most accurate descriptor for biochemical assessment of treatment outcome in acromegaly and was adopted at this 14th Acromegaly Consensus Conference (SR).

Importantly, although biochemical remission is the primary assessment of treatment outcome, it is not the only goal of treatment in acromegaly. In all cases, biochemical findings should be interpreted within the clinical context of acromegaly signs and symptoms (SR). Maintaining serum IGF-I level in the mid to upper half of the age-related reference range could be considered in clinically controlled patients to avoid induction of GH deficiency [64] (HQ).

### Postoperative remission

There are no definitive studies on the optimal assessment for postoperative remission, nor of the timing of its evaluation. Remission rates after surgery using OGTT results are influenced by the defined cutoff for GH normalization, timing of measurement, and adenoma characteristics. For example, some studies reported approximately 60% of patients achieve biochemical remission in the immediate postoperative period when defined as nadir GH < 1 µg/L during OGTT, with lower rates in patients with macroadenomas and in those treated with a microscopic approach [65, 66] (MQ). However, remission rates fell to approximately 40% when using stricter criteria of < 0.4 µg/L on postoperative day 2, and 20% of patients achieved GH below threshold after a delayed period of a median of 24 months of observation [67] (LQ). Of note, very early (and tighter) GH control might be predictive of later GH outcome, as nadir GH > 0.4 µg/L on postoperative day 2–5 predicted lack of remission after a mean follow-up of 44 months [68], and nadir < 0.4 µg/L at 2–5 days and at 3–6 months correlated better with remission than did < 1 µg/L [68, 69] (LQ).

Generally, IGF-I normalization measured 12 weeks after surgery defines surgical success [5, 70] (SR). Some studies defined early remission as normalization at 6 weeks [71], while others included patients who achieved remission after 12 weeks [72], or up to 12 months after surgery [73]. However, delayed IGF-I normalization has been seen as late as 24–57 months after surgery [74, 75]. When measuring random GH, studies have used gradually decreasing normal cutoffs, moving from < 3 to < 2 µg/L and ultimately to < 1 µg/L, with reported remission decreasing accordingly from 89–99% to 61–79% [76–79] (LQ). These studies showed that early postoperative assessment at 1 day after surgery predicted long-term remission, and others have confirmed that elevated random GH on postoperative day 1 or 2 strongly predicts persistent disease [80] (LQ).

Although significant age and sex differences in postoperative GH levels have been noted [81] (VLQ), population-specific thresholds for remission have not been established.

Discordant GH/IGF-I results may be an indicator of mild ongoing disease activity, reflecting dysregulated but persistent somatotroph GH secretion and tissue responsiveness [82, 83]. Discordant GH/IGF-I results may also reflect a delay in IGF-I return to normalization after surgery [70, 74], potentially determined by GH receptor polymorphism [84]. However, it may also be a function of assay variability and changed cutoffs of normal results (VLQ). In a meta-analysis of > 7000 patients evaluated over a 25-year period, 26% showed discordant GH/IGF-I when using a GH nadir cutoff of < 1 µg/L, while 31% showed discordance when using a cutoff of < 0.4 µg/L [85] (MQ). Evaluating GH levels from the mean of 3 consecutive assessments using the same validated assay rather than a single assessment can lessen the impact of GH cutoff on discordance [86] (VLQ).

IGF-I levels should be measured 12 weeks after surgery to determine postoperative biochemical remission (SR). As the magnitude of GH decrease in the immediate postoperative period likely reflects the degree of success in adenoma removal, early random GH assessment on day 1–14 and comparison with preoperative GH levels can inform the degree of adenoma removal and subsequent longer-term remission (DR). OGTT assessment may provide further predictive value (DR). As preoperative SRL, used in patients with risk factors for more adverse surgical outcomes [87], may have carryover effects that continue to influence postoperative IGF-I values [88] (MQ), assessment should be repeated at 3–6 months to confirm remission (DR).

### Remission with adenoma-directed medical therapy

Long-term follow-up of patients with acromegaly shows no increase in mortality risk in patients who maintain normalized IGF-I [89] (MQ), and improved rates of biochemical control in more recent years has been attributed, at least in part, to effective GH suppression with use of SRL therapy [90, 91] (LQ). However, as injectable SRL is administered monthly, timing of assessment for IGF-I could influence determination of biochemical control. In one study [92], wide variability in IGF-I levels was seen upon weekly assessments in patients treated with long-acting octreotide or lanreotide, but not in acromegaly patients in continued postoperative remission not treated with SRL or in healthy controls (LQ). At least one IGF-I level ≥ 2 standard deviations above normal was seen in 10–20% of patients during the treatment cycle. As the last sampling just before the next injection was the best predictor of variability [92], consistent with the waning of QOL seen at the end of the treatment cycle [93] (LQ), this is the recommended timing for IGF-I assessment during injectable SRL therapy (DR). IGF-I level measured in the last week before the next injection should therefore be used to determine a need for dose titration or consideration of alternative treatment options if normalization is not achieved (SR).

For patients treated with oral SRL administered daily, assessment of IGF-I for the purposes of dose titration should be done after at least 2 weeks of treatment (SR) [94]. Timing of IGF-I assessment is not critical for patients treated with cabergoline administered in more than once-weekly intervals; the timing of assessment for patients treated once weekly has not been systematically investigated (VLQ).

With all of these agents, random GH assessment is not likely to provide additional information in all patients, but could be considered for symptomatic patients with IGF-I levels at the higher end of the ULN (DR).

### Remission with peripherally directed medical therapy

In clinical trials of the GH receptor antagonist pegvisomant as first-line medical therapy, 82–92% of patients achieved normalized IGF-I [95] (HQ). Real-world studies of pegvisomant used mostly as second- or third-line medical therapy show approximately 54–64% of patients maintain biochemical control over the long term [96, 97] (MQ). Lower rates are likely due, at least in part, to inadequate dose titration [98]. Nevertheless, regardless of IGF-I control, patients showed consistent improvements in QOL [99] (LQ) as well as decreased blood glucose in those with and without diabetes [100] (LQ), suggesting that suppression of peripheral GH action has a broader effect on disease activity beyond IGF-I control.

Estrogens and selective estrogen receptor modulators (SERMs) inhibit hepatic IGF-I production but currently have a limited role in acromegaly management [101, 102] (VLQ).

For patients treated with medical therapy that targets the GH receptor or the estrogen receptor, efficacy assessment is limited to IGF-I normalization (SR). With these agents, GH assessment is not informative and should not be performed.

## Follow up

Acromegaly is a chronic disease, requiring lifelong monitoring to prevent or minimize deleterious effects of GH hypersecretion. Yet, acromegaly is also a heterogenous disease, and complex treatment algorithms describe multiple potential monotherapy and combination therapy approaches depending on individual patient and adenoma characteristics [17]. Follow up assessments should therefore consider biochemical evaluation of treatment effectiveness, imaging studies evaluating residual or recurrent mass, and clinical signs and symptoms of acromegaly complications and comorbidities (SR).

### Biochemical assessments

Multiple groups have considered optimal timing for GH/IGF-I assessment in determining postoperative remission. However, there is currently no known optimal timing for continued biochemical assessment (VLQ). Given the high rates of biochemical remission after microadenoma resection [103] (HQ), and the very low rates of recurrence among those who achieve postoperative remission even after 10 years of follow up [104, 105] (MQ), rigorous studies to define optimal assessment timing are likely not feasible.

Within the first postoperative year, IGF-I measurements every 3–6 months may be appropriate to confirm remission and then every 6–12 months to monitor for potential recurrence (SR). OGTT might be helpful in evaluating patients with borderline IGF-I levels and clinical signs of disease activity (DR).

For patients who did not achieve postoperative remission and who are treated with adjuvant SRL, IGF-I should be assessed 3 months after initiation/dose adjustment of injectable SRL and 2–4 weeks after initiation/dose adjustment of oral SRL to establish an optimal dosing regimen [94], and then every 6–12 months thereafter once biochemical control is achieved (SR). Random GH might be helpful in select cases where evaluation of adenoma behavior is a concern (DR). As pegvisomant and cabergoline have a shorter half-life than injectable SRL, IGF-I should be assessed every 1–3 months after treatment initiation/dose adjustment to establish the dosing regimen, and then every 6–12 months thereafter. GH assessment is not informative in follow-up of pegvisomant and cabergoline and should not be performed (SR).

In patients receiving radiotherapy to the residual/recurrent mass, medical therapy is used as a bridge until radiation effect is seen [106]. In these patients, IGF-I should be assessed at the intervals appropriate for the medical therapy used (SR). With sustained decline of IGF-I within the target range, treatment can be paused at least once each year depending on rapidity of the IGF-I decline to test for the onset of radiation-induced remission (DR).

For all patients, ideally, the same well-validated IGF-I assay should be used for all assessments (SR). New or persistent elevations in IGF-I levels should be interpreted within the context of the individual clinical scenario and account for factors that could affect results such as pregnancy, estrogen use, starvation, and metabolic changes (SR).

### Imaging studies

Long-term follow up of patients who achieve postoperative biochemical remission show that a vanishingly small percentage of those with recurrence show evidence of new tissue mass on MRI, and fewer still require a second surgery [104] (LQ). Similarly, while SRL use can result in adenoma shrinkage in approximately one-third of patients, particularly when used as primary medical therapy [107–109] (HQ) adenoma *growth* during SRL therapy is rare [110–112] (VLQ), and it is likely that such patients would first demonstrate biochemical changes if they recur. Even in patients treated with pegvisomant, after 14 years of follow up, central reassessment of equivocal MRIs led to only 1.4% of patients discontinuing treatment due to adenoma growth [96] (MQ).

Therefore, regular MRI follow-up is not indicated for all patients with acromegaly (SR). Patient-specific factors informing the need for follow up MRI (DR) include those older at presentation who are more likely to have smaller adenomas and less aggressive disease [113] (VLQ), and those with T2-weighted hypointensity who are more likely to demonstrate more favorable SRL responsiveness [41] (LQ), suggesting that these cohorts are less likely to exhibit clinically relevant adenoma re-growth on MRI.

The same standards for imaging and results reporting should be used in follow up as in diagnosis (SR). MRI should be performed at 3–6 months postoperatively and used as baseline for further assessments (SR). Thereafter, MRI should be performed upon signs of biochemical or clinical disease progression, and when a change in therapeutic modality is considered, such as prior to a second surgery or radiotherapy (SR). ^11^C-methionine PET imaging may aid localization of residual adenoma in patients with persistent GH hypersecretion following primary (and subsequent) therapy when MRI findings are equivocal [45, 46] (DR). An individualized approach to MRI is appropriate for patients treated with pegvisomant based on country-specific labeling requirements, as well as for those with genetic syndromes or prior imaging suggestive of highly aggressive disease (DR).

### Clinical assessments

Effective management of acromegaly disease comorbidities and complications over the long term is critical to maximizing patient outcomes. Although cardiovascular disease is no longer the leading cause of mortality in patients with acromegaly [114, 115] (MQ), hypertension and diabetes are associated with increased cardiovascular morbidity and mortality [116, 117] (MQ). This Workshop endorsed evaluation and treatment of disease comorbidities according to prior consensus recommendations [8] (SR). The need for assessment of common comorbidities, such as hypopituitarism, obstructive sleep apnea, and vertebral fractures depends on clinical symptoms and adenoma behavior, and follow up according to accepted guidelines [8, 118] was recommended (SR).

Although acromegaly patients are at increased risk for colon cancer, increased rates of cancer-specific mortality have not been shown [119] (MQ). Guidelines for screening high-risk patients from the British Society of Gastroenterology and Association of Coloproctology for Great Britain and Ireland suggest regular screening beginning at age 40, and individualized considerations for repeat colonoscopy according to evidence of acromegaly disease activity and prior colonoscopy findings [120]. Nevertheless, there was no consensus at this Workshop on whether colonoscopy should be performed in all patients at diagnosis of acromegaly regardless of age, despite the discretionary recommendation in previous consensus publications [8]. For all other cancers with reported increased frequency in acromegaly, including thyroid cancer, there was consensus that screening be performed according to national/regional guidelines for the general population [8].

### Tools for assessment

SAGIT and ACRODAT are scoring tools that use multiple disease-specific parameters to define severity of acromegaly [121, 122]. With SAGIT, clinicians have the opportunity to standardize scoring to evaluate signs and symptoms, associated comorbidities, GH levels, IGF-I levels, and adenoma characteristics. Although results from the validation study showed that IGF-I and GH levels drove disease activity scoring, non-biochemical indicators of disease activity influenced treatment decisions [121 (MQ)]. With ACRODAT, clinicians rate disease activity as stable, mild, or severe based on IGF-I level, adenoma status, comorbidities, symptoms, and QOL, and the validation study showed that elevated IGF-I and evidence of adenoma growth drove definition of disease severity [122] (MQ).

Both instruments may be useful in clinical practice for assessing changes in acromegaly disease severity and progression over time (DR). A prospective study measuring a clinically beneficial effect of ACRODAT as a monitoring tool is underway.

### Considerations for selecting second and third-line medical therapy

Follow up assessments identify patients more likely to show a good response to second- and third-line medical therapy options if needed. For example, among patients unresponsive to octreotide/lanreotide, those with T2 MRI hyperintensity are more likely to show improved IGF-I levels while receiving pasireotide (VLQ), and those with lower SST2 and higher SST5 receptor expression are more likely to achieve adenoma shrinkage with pasireotide [123] (LQ). By contrast, those with lower SST5 receptor expression are less likely to respond to pasireotide, and those with adenoma extension to the third ventricle are less likely to respond to both pasireotide and pegvisomant [124] (LQ). For patients demonstrating insufficient control on single-agent adenoma-targeting SRL, the addition of a peripherally targeting GH receptor antagonist could be beneficial; in such cases, the combination of low-dose SRL plus weekly pegvisomant is both highly effective and more cost-effective than higher doses or more frequent dosing of these agents [125] (MQ).

Results of follow up assessments can also be used to identify patients who might benefit from treatment options that have an improved safety profile or more convenient dosing regimen (SR). For example, pegvisomant can improve metabolic outcomes [126] (MQ), which may be indicated for patients demonstrating glycemic changes with SRL monotherapy (DR). Pasireotide might have a more effective shrinkage effect than octreotide and lanreotide [109] and may be indicated in patients with clinically relevant residual adenoma mass (DR). Oral octreotide has proven effective in maintaining biochemical control in patients previously controlled on octreotide LAR or lanreotide depot injection therapy [127] (HQ). The side effect profile is similar to that of octreotide LAR even when used at the highest doses, yet data from extension trials show that more patients prefer oral over injectable administration [128], and such an option could be considered to address QOL concerns (DR).

## Conclusions

Acromegaly is an insidious disease with potential lethal consequences if not diagnosed and treated in a timely manner, as is unfortunately commonly reported. In this context, therapeutic inertia, as for other chronic diseases, is also frequently manifest. Therefore, the outcomes of the 14th Acromegaly Consensus Conference are particularly clinically relevant. The current statement updates and refines previous statements from our Group concerning the proper approach to diagnose acromegaly using biochemical, clinical, and imaging criteria. Moreover, the current statement also includes new recommendations on assessment of “remission” after each specific treatment tool. Recommendations on the optimal follow-up of acromegaly both in terms of timing and methodologies are presented. Worldwide application of the current recommendations should improve management of acromegaly, helping, at least in part, to mitigate the adverse impact of commonly observed diagnostic delay and therapeutic inertia.

## Data Availability

Not applicable.
