# Correction: consensus on criteria for acromegaly diagnosis and remission

**DOI:** 10.1007/s11102-023-01373-w

**Published:** 2023-12-06

**Authors:** Andrea Giustina, Nienke Biermasz, Felipe F. Casanueva, Maria Fleseriu, Pietro Mortini, Christian Strasburger, A. J. van der Lely, John Wass, Shlomo Melmed, Acromegaly Consensus Group

**Affiliations:** 1 San Raffaele Vita-Salute University and IRCCS Hospital, Milan, Italy; 2 Leiden University Medical Center, Leiden, The Netherlands; 3 Santiago de Compostela University, Santiago de Compostela, Spain; 4 Oregon Health and Science University, Portland, OR USA; 5 Charité Universitätsmedizin, Berlin, Germany; 6 Erasmus University Medical Center, Rotterdam, The Netherlands; 7 Churchill Hospital, Oxford, UK; 8 Cedars-Sinai Medical Center, 8700 Beverly Blvd, NT 2015, Los Angeles, CA 90048 USA

**Correction: Pituitary**

10.1007/s11102-023-01360-1.

In this article the statement in the Funding information section was incorrectly given as 'The 14th Acromegaly Consensus Conference was supported by unrestricted educational grants from Amolyt Pharma, Amryt Pharma, Basecamp Bio, Crinetics Pharmaceuticals, Ionis Pharmaceuticals, and Recordati Rare Diseases’ and should have read The 14th Acromegaly Consensus Conference was supported by unrestricted educational grants from Amolyt Pharma, Amryt Pharma, Basecamp Bio, Crinetics Pharmaceuticals, Ionis Pharmaceuticals, Novo Nordisk, Pfizer, and Recordati Rare Diseases. The original article has been corrected.

